# Temporal Context Influences the Perceived Duration of Everyday Actions: Assessing the Ecological Validity of Lab-Based Timing Phenomena

**DOI:** 10.5334/joc.4

**Published:** 2018-01-09

**Authors:** Nadine Schlichting, Atser Damsma, Eren Erdal Aksoy, Mirko Wächter, Tamim Asfour, Hedderik van Rijn

**Affiliations:** 1 Department of Psychology, University of Groningen, Groningen, NL; 2 Institute for Anthropomatics and Robotics, Karlsruhe Institute of Technology, Karlsruhe, GE

**Keywords:** Time perception, Memory, Event cognition

## Abstract

Timing is key to accurate performance, for example when learning a new complex sequence by mimicry. However, most timing research utilizes artificial tasks and simple stimuli with clearly marked onset and offset cues. Here we address the question whether existing interval timing findings generalize to *real-world* timing tasks. In this study, animated video clips of a person performing different everyday actions were presented and participants had to reproduce the main action’s duration. Although reproduced durations are more variable then observed in laboratory studies, the data adheres to two interval timing laws: Relative timing sensitivity is constant across durations (*scalar property*), and the subjective duration of a previous action influenced the current action’s perceived duration (*temporal context effect*). Taken together, this demonstrates that laboratory findings generalize, and paves the way for studying interval timing as a component of complex, everyday cognitive performance.

## Introduction

Timing and the perception of time are fundamental aspects of our daily life. Anything we perceive and experience is expressed over time, and adaptive cognitive performance requires accurate timing, ranging from determining the time between glances in the rear-view mirror while driving, to a well-timed pause in a speech to increase rhetorical effectiveness. The timing of short intervals, referred to as interval timing, has been extensively studied. However, these studies typically entail processing an interval marked by simple and static stimuli with highly salient and clearly defined on- and offsets. This leaves no ambiguity about the exact start- and endpoint, whereas events in the real world often lack sharp and salient on- and offsets, as, for example, it is not clearly defined at what sound level a silence commences. In addition, unlike the focused setting of experimental studies, interval timing in the real world is usually embedded in a context consisting of many different durations associated with different subtasks perceived from the first- or third-person perspective. As a consequence, results from laboratory studies might have low external validity as it is unclear to what extent the general “laws” derived from extensive laboratory findings generalize to more complex environments (Darlow, Dylman, Gheorghiu, & Matthews, 2013; Matthews & Meck, 2014; van Rijn, 2014). Here, we report on a study that addresses the question of whether the two prominent law-like properties, scalar property and context effects, generalize to the timing of more realistic timing tasks.

The *scalar property* (e.g., Allman, Teki, Griffiths, & Meck, 2014; Wearden & Lejeune, 2008; for an extensive review, see Grondin, 2014) is a form of Weber’s law, stating that the standard deviation of repeated estimations of a duration is proportional to the average estimated duration. Consequently, timing sensitivity follows Weber’s law in that relative sensitivity is preserved for varying interval durations.

The second phenomenon is the effect of context on interval timing. Context effects can be found on a global level (Vierordt’s law) and a more local level (trial-by-trial effects). Vierordt’s law describes the central tendency effect: when confronted with varying interval durations, participants tend to overestimate short durations and underestimate long durations (Lejeune & Wearden, 2009). By manipulating temporal context, Jazayeri and Shadlen (2010) showed that participants under- or over-reproduced the duration of the same interval depending on temporal context, and explained their findings using a Bayesian model which takes into account the underlying distribution of samples. Local context effects are more concerned with the recent history of encounters with intervals of varying durations (i.e. sequential dependencies). Dyjas, Bausenhart and Ulrich (2012) explained sequential effects and fluctuations in behavioural performance with a mathematical model which, on each trial, combines previously and currently available information about stimulus durations to form one representation. Both global and local contexts effects have been extensively studied not only in laboratory-based timing tasks (see van Rijn, 2016, for a review), but also in non-timing tasks (e.g., distance, length or angle estimations as reviewed in Petzschner, Glasauer, & Stephan, 2015), showing that these effects are highly robust and apply to many psychophysical tasks.

As stated above, these psychophysical laws have been established in artificial experimental settings. As a first step towards the use of ecologically valid stimuli a new line of research has emerged in which more dynamic stimuli are used (e.g., moving or rotating geometrical shapes; Matthews, 2011; Sasaki, Yamamoto, & Miura, 2013). Interestingly, this work demonstrated that using dynamic stimuli affects the subjective perception of time, as moving stimuli seem to last longer than static ones, and apparent duration increases with increasing stimulus speed, a phenomenon known as subjective dilation (Eagleman, 2008). These results thus indicate that findings observed in the traditional literature might be specific to simple, static stimuli, and not generalize to more realistic stimuli. This suggestion is supported by results of a “real-world” experimental paradigm in which the effect of speed of driving on time perception was estimated (van Rijn, 2014). Video snippets from a recording of a driving simulator session served as stimuli (driver’s perspective), and were played at either original, faster or slower speed. The data exhibited the same subjective dilation effects previously found (Eagleman, 2008): perceived duration increased with increasing driving speed. Interestingly, this study did not just demonstrate that previous findings on subjective dilation generalize to real-world-like settings, but also demonstrated that laboratory findings in which typically perspective is not manipulated generalizes to timing from a first-person perspective.

In fact, perspective seems to be a highly understudied factor — although perspective is an important aspect of being in the real world. Everything we perceive, and thus also time, is perceived from first-person perspective. With simple stimuli the effect of perspective cannot be tested because there is no change in perspective possible. Therefore, the effect of perspective on time perception is as of yet unknown. However, it has been shown that perceived or imagined distance affects time perception, in that events that happen further away from the observer seem to last longer (e.g., Gorea & Hau, 2013; Zäch & Brugger, 2008). One could imagine that first-person events happen spatially closer to the observer than third-person events — in line with the findings discussed above, perspective could alter time perception (in-)directly.

Yet another difference between timing static or moving shapes in laboratory studies and timing in real-life settings is that ecological timing is often part of human-object- or human-human-interactions. Studies focusing on differences in time perception between animate and inanimate figures have shown that animacy affects time perception (Carrozzo & Lacquaniti, 2013; Carrozzo, Moscatelli, & Lacquaniti, 2010; Orgs, Bestmann, Schuur, & Haggard, 2011). This work led to the proposition that different mechanisms underlie the timing of biological motion (animate stimuli) and visual motion (inanimate stimuli), providing additional arguments against generalizing results from traditional laboratory studies to real-life timing contexts (Lacquaniti et al., 2014). Further, these studies sketch interval timing as a multifaceted process, in which perspective and the nature of the action that needs to be timed could have significant impact on perceived duration. For example, Garsoffky, Huff and Schwan (2017) showed that, depending on semantic temporal gaps in videos showing series of everyday actions performed by human actors, the duration of a specific action was under- or overestimated. Videos in the long temporal gap condition consisted of clips of temporally distant actions and the target action was systematically underestimated, while in the short temporal gap condition videos consisted of clips of actions that are temporally closer together in the stream of events and caused the target duration to be overestimated in duration.

**Figure 1:**
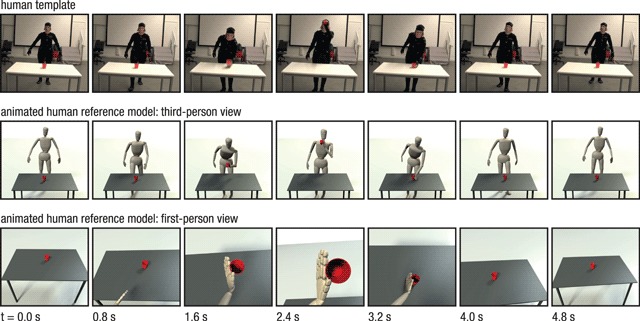
Exemplary depiction of the action *drink* in the *short* video condition. Animated videos were obtained from videos of an actress doing the same everyday actions with real objects and wearing a motion capture suit (top). The two bottom panels show the animated human reference model as seen from third- (middle) and first-person perspective (bottom) based on the human template video.

In the current study, we aimed to investigate interval perception in naturalistic contexts, while retaining optimal experimental control. Specifically, we asked whether common effects found in traditional laboratory studies, the scalar property and context effects, can be generalized to timing in real-world settings. Furthermore, we aimed to explore possible effects of perspective on time perception. To this end, participants were asked to reproduce the duration of an action demonstrated in a video. Each video showed an animated person performing six different everyday actions filmed from first- or third-person perspective (for an example, see Figure 1). After watching the video participants were asked to reproduce the duration of the action (e.g., “drinking”) by pressing a key. Importantly, participants were not explicitly instructed when to start or end timing.

## Materials and Methods

### Participants

Twenty-three students enrolled in the Psychology programme of the University of Groningen (mean age: 19.3 years, range: 18–22 years, 17 female) participated in the experiment and received partial course credit. Sample size was based on past research (e.g., 12 participants in Carrozzo & Lacquaniti, 2013; 38 participants in van Rijn, 2014). All participants had normal or corrected-to-normal vision. Informed consent as approved by the Psychology Ethical Committee of the University of Groningen (15008-NE) was obtained before testing.

### Stimuli

Videos of an animated human reference model (see below for details) performing six different actions recorded from third- and first-person perspectives and performed over shorter or longer durations served as stimuli. The actions were *drink* (figure drinks from a cup), *mix* (figure uses a whisk to mix something in a bowl), *pour* (figure pours a liquid into a cup), *put on* (figure picks up a bowl and puts it on another object), *take down* (figure picks up a bowl placed on another object and puts it down on the table), and *pick & place* (figure picks up a whisk and puts it down at a different location on the table). All action-related objects were located on a table before and put back on the table after the actions were performed. Importantly, video length was not correlated with the speed of the action (e.g., drinking fast), but video length determined how long an action, performed at normal speed, was executed.

Animated videos were based on video recordings of a human actress performing each of the six actions over different time spans (Figure 1, top row). Movements were recorded by a marker-based motion capture system (VICON, http://www.vicon.com, sampling rate of 100 Hz, marker position accuracy < 1 mm). Human movement recordings were encoded using the Master Motor Map (MMM) toolkit (Terlemez et al., 2015), which is underlying a generic framework for unified representation and transfer of whole-body human motions to different embodiments (e.g., humanoid robots or animated characters). Finally, the scene and the animated human reference model were generated with MMM-based animated characters and 3D object models (e.g., a red cup) as described in Wächter and Asfour (2015) and illustrated in Figure 1. The entire motion data set, including MMM representations and object models, is publicly available as a part of the KIT whole-body human motion database (available at: https://motion-database.humanoids.kit.edu/) (Mandery, Terlemez, Do, Vahrenkamp, & Asfour, 2015).

We chose to use an animated human reference model instead of human actors to control for confounding factors like variations in background noise or emotions conveyed by facial expressions (e.g., Fayolle & Droit-Volet, 2014). With 6 actions, 2 perspectives, and 2 length condition, a total of 24 animated videos were created to be used as stimuli.

### Procedure

Participants were seated, at normal operating distance, in front of a Iiyama Vision Master Pro 513, 22 inch screen with a resolution of 1024 × 768 px and a refresh rate setting of 100 Hz. Participants were provided with instructions informing them that they would be asked to reproduce the duration of an action observed in the video presented on that trial. Each experimental trial started with the presentation of a short text describing the action that would be presented (e.g., “mixing”). After 3 s, one of the 24 videos was presented centrally with a resolution of 960 × 540 pixels. After the end of the video the screen went blank for 1 s, followed by a grey circle presented in the centre of the screen. This circle indicated that participants could reproduce the duration of the action by pressing down the spacebar. As a visual aid, the grey circled turned white while the spacebar was pressed down (i.e. during the interval reproduction). One additional trial sampled randomly from the 24 available videos served as a practice trial at the beginning of the experiment. Data from this trial were not analysed. Importantly, no explicit instructions (or visual or auditory aids) were provided to indicate when an interval was supposed to start or end; the start- and endpoints of a specific action had to be determined by the participants themselves. However, it was explicitly stated in the instructions that the start- and endpoint of the action would not necessarily coincide with the start and end of the video. Videos were presented in random order. Each video was presented four times, resulting in a total of 96 trials. The experiment was programmed in OpenSesame version 3.0.3 (Mathôt, Schreij, & Theeuwes, 2012). The experimental script is available online at: http://osf.io/y9zex.

### Objective Durations

To compare the reproduced durations to objective durations, we computed objective durations using the Semantic Event Chain (SEC) extraction algorithm that is used in AI and robotics to decompose actions or action sequences into atomic (sub-)actions (Aksoy, Aein, Tamosiunaite, & Worgotter, 2015; Wächter & Asfour, 2015). During SEC extraction, videos are examined and segmented according to spatiotemporal hand-object relations and actions can be categorized (e.g., *approach, grasp, withdraw*) based on semantic object-object and object-hand contact relations. Based on this segmentation process, we defined the duration of the main action as lasting from the first contact with the involved object until letting go of that object, resulting in the following durations (ordered from shortest to longest): 0.83s (take down short version), 0.96s (put on short), 1.07s (pick & place short), 1.82s (pick & place long), 1.88s (put on long), 2.32s (drink short), 3.02s (take down long), 3.24s (mix short), 3.68s (pour short), 6.63s (mix long), 8.06s (pour long), 8.92s (drink long). We validated this algorithm in an additional experiment (see Supplemental Material available at: https://osf.io/y9zex).

### Data Analysis

The data analyses focused on three main questions: (1) Do the local and global context influence the perceived duration; (2) Is there an effect of perspective on perceived duration; (3) Is the scalar property observed in these data, operationalized by two different measures discussed below. For these analyses, we created Linear Mixed Models (LMMs) using the *lme4* package (version 1.1–10; Bates, Mächler, Bolker, & Walker, 2014) in R version 3.2.2 (R Development Core Team, 2008). To test whether including a fixed factor improved the LMM, we performed model comparisons using likelihood ratio tests. Participant and action were always entered as random intercepts. After model comparison, we sequentially added random slopes for participant and the fixed factors, and action and the fixed factors (excluding objective duration), to the model to test whether this affected the results. In all LMM analyses, we found that the random slopes did not change the effects of the fixed factors qualitatively. Therefore, we will report the results of the simpler random intercept models here. For both significant and non-significant fixed factors in the LMMs, we used Bayesian analyses to quantify the evidence in favour of the alternative hypothesis. To this end, we compared the model including the fixed factor with the model without the factor using the *lmBF* function from the *BayesFactor* package in R (Morey, Rouder, & Jamil, 2014). The evidence for H_1_ over H_0_ will be denoted as BF_10_. All analysis scripts and data are available at: http://osf.io/y9zex.

First, we tested the data for context effects. To test whether there was a global pull towards the (subjective) mean, an LMM was estimated with bias (the difference between the reproduction and the objective duration) as dependent variable and objective lag as fixed factor. Whereas perfect reproduction would yield a bias that is consistently 0 over durations, a pull towards the mean would result in a negative effect of duration on bias. To investigate local context effects, we tested the effect of the previous (n-1, n-2, …) *subjective* duration (i.e. the previous produced duration) and the previous *objective* duration (i.e. the objective duration of the action, as provided by the action segmentation algorithm) on the current trial (i.e. the current produced duration). Reproduced action duration was entered as dependent variable. Objective duration of the current trial, perspective, and previous subjective or objective trial duration were entered as predictors. Following Taatgen and van Rijn (2011), we iteratively compared more complex models (e.g., a model including n-3, n-2, n-1) with simpler models (e.g., a model including n-2 and n-1). The same model comparison approach was used to test for effects of perspective.

To test the data for the scalar property, following Wearden and Lejeune (2008), the coefficient of determination (*r*^2^) was determined by correlating the standard deviation of the reproduced action duration (*S*), averaged over all participants, with the mean reproduced duration (*M*) of each action in the long and short condition separately (i.e. for the 12 unique action-duration combinations, data from the different perspectives was pooled together). Additionally, the coefficient of variation (*CV* = *S* ÷ *M*) was calculated for each video and participant separately. An LMM was constructed to assess the influence of duration on *CV*, testing whether *CVs* differed across action durations.

## Results

**Figure 2:**
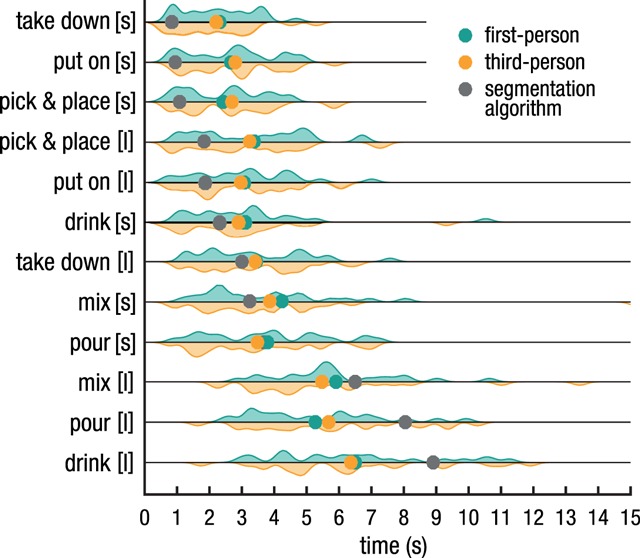
Estimated action durations. Coloured dots indicate mean reproduced durations for each action (short indicated with [s], long with [l]), averaged over all participants. Violin plots illustrate the density distributions of participants’ reproduced action durations. Grey dots depict the objective duration as defined by the action segmentation algorithm. Generally, shorter durations were overestimated and longer durations underestimated, demonstrating typical context effects.

**Figure 3:**
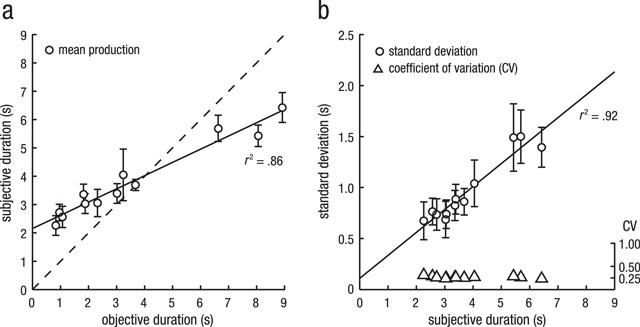
Estimated action durations **(a)** and variance associated with estimated durations **(b)** for the short and long version of the 6 different actions. Panel A depicts mean reproduced durations (error bars indicate the 95% CI, corrected for between-subject variability), averaged over all participants and first-/third-person perspective trials, plotted against the objective duration. Reproduced durations differ from the objective durations (diagonal dashed line): shorter durations were overestimated, whereas longer durations were underestimated, demonstrating context effects. Panel B depicts the standard deviation over subjective duration for all 12 actions. Conforming to the scalar property, regression of mean standard deviation (error bars indicate the 95% CI, corrected for between-subject variability) against mean reproduced duration (circles) revealed a linear relationship (solid line) with *r*^2^ = .92. Calculated coefficients of variation (triangles) did not differ significantly from each other.

Figure 2 and Figure 3A depict the mean reproduced action durations for each action in the short and long version (i.e. for each objective duration). Visual inspection of the results in Figure 2 suggests that shorter durations were overestimated, whereas longer durations were underestimated. This can be seen in Figure 2 as the reproduced durations (green/orange dots) for the shorter durations (at the top of the figure) were reproduced as longer than the objective durations (grey dots), whereas the inverse is observed for the long action duration (at the bottom of the figure). Indeed, the LMM showed that the reproduction bias decreased with duration (β = –0.51, *t* = –31.06, *p* < .001, BF_10_ > 100), indicating a significant pull towards the (subjective) mean.

If this pull towards the mean is driven by a continuously updating memory representation that reflects recent experiences, the durations perceived before the current trial must influence the current trial’s estimate. Model comparisons showed that including subjective N-1 improved the model predicting reproduced action duration significantly (χ^2^ = 66.19, df = 1, *p* < .001, BF_10_ > 100). Thus, the subjective duration estimation in the previous trial influenced the duration estimation of the current trial (β = 0.13, *t* = 8.23, *p* < .001), with previous estimates being positively correlated with the current estimate. Adding N-2 to the model did not improve the goodness of fit (χ^2^ = 2.08, df = 1, *p* = .149, BF_10_ = 0.11). For theoretical reasons, and following Taatgen and van Rijn (2011), we did not test for an influence of N-3 without including N-2. These results support the hypothesis that reproduced action durations deviate systematically from the objective durations, with a pull towards the mean.

Alternatively, however, the effect of the previous trial on the current reproduction could potentially be explained by attentional fluctuations or performance drift over the course of the experiment. It could, for example, reflect a change in the willingness of the participant to hold down the response key. To disentangle the influence of the subjective N-1 and these overall performance fluctuations, we calculated the relative bias (i.e. the difference between the reproduced and objective duration divided by the objective duration) for each trial. If the N-1 effect depends on performance fluctuations, we would expect that the bias in the current trial depends on the bias in the previous trial (e.g., if the estimation in the previous trial was too short, it will also be too short in the current trial). If, however, the N-1 effect depends on the actual subjective duration in the previous trial, we would expect that the current bias reflects the magnitude of this subjective duration (e.g., a negative bias if the previous subjective duration was shorter than the current duration). We created a mixed model with bias as the dependent variable, and objective duration, previous subjective duration and previous bias as fixed factors. Model comparison showed that both previous subjective duration (χ^2^ = 44.39, df = 1, *p* < .001, BF_10_ > 100) and previous bias (χ^2^ = 9.28, df = 1, *p* = .002, BF_10_ = 5.59) improved the model significantly. Both previous subjective duration and previous bias contributed positively to the current bias (β = 0.06 and β = 0.05, respectively). Thus, whereas performance drift seems to play a role, it does not account for the effect of the previous reproduction on the current reproduction.

We also tested for the effect of the *objective* N-1. In contrast to the subjective N-1 duration, we found that adding the objective N-1 duration did not improve the model (χ^2^ = 2.77, df = 1, *p* = .096, BF_10_ = 0.11). Thus, it is not the objectively observed duration of the previous trial that influences the current trial’s estimate, but how this previous duration is observed.

As might be expected based on the similar locations of the orange and green dots in Figure 2, including perspective as a predictor of reproduced duration did not improve the model’s fit (χ^2^ = 1.90, df = 1, *p* > .250, BF_10_ < 0.01). Supported by a decisive Bayes Factor, it can be concluded that manipulating whether participants saw an action in first- or third-person perspective did not influence perceived durations in the current experimental setup. Consequently, data was pooled over perspective for subsequent analyses.

To assess whether the scalar property held in the current data, we addressed the correlation between the standard deviations and the mean reproduced durations and tested whether subjective duration predicted coefficients of variation. The results of both analyses suggest that the scalar property holds, as the standard deviations (*S*) and mean reproduced durations (*M*) were highly correlated with *r*^2^ = .92, *p* < .001 (Figure 3B) and the *CV* values of the different action durations did not differ from each other, as revealed by an LMM performed on the *CV* values of all participants (β = 0.00, *t* = –0.95, *p* > .250, BF_10_ = 0.23).

## Discussion

Do the general laws of interval timing, derived from scores of experiments using static and highly artificial stimuli, generalize to more real-world like settings? Although some studies using dynamic stimuli suggest that interval timing can be affected when more realistic stimuli are used, this study demonstrates that two basic time perception laws hold, even when naturalistic stimuli are used. These results pave the way for integrating interval timing theories in computational or quantitative theories of complex cognitive behaviour studied in real-world settings. For example, when operating complex machinery, many intervals need to be tracked simultaneously as different aspects of complex machines may need attention at different intervals, or when speaking in front of a group of individuals, specific intervals might be associated with different aspects of interaction. This research demonstrates that in these cases, the global context effect will cause overestimation of the shortest duration and underestimation of the longest, local context effects predict sequential dependencies on a trial-by-trial basis, and the scalar property suggests that the error in shorter duration will be smaller than the error in longer durations. Taking these effects into account can explain additional variance when studying complex tasks, providing researchers with a more direct picture of the non-temporal aspects of the task under study.

Before turning to the effect of context and the scalar property, one aspect of the empirical data needs to be discussed as it deviates from the results obtained with more standard stimuli. As can be seen in Figure 3, Panel B, the average coefficient of variation is around .25, and the regression line fitted through the average standard deviations and subjective durations has a slope of .23. These values are notably larger than reported in a review of coefficients of variations by Gibbon, Malapani, Dale and Gallistel (1997). In this review, durations between 2 and 8 seconds have an average coefficient of variation below .2, often closer to .1. This difference can also be observed by comparing the probability densities of interval reproductions of the current study (Figure 2) with a laboratory-based reproduction task using auditory stimuli with clear on- and offsets (Figure 2 in Kononowicz, Sander, & Van Rijn, 2015), as the artificial stimuli resulted in narrower distributions. A major difference is that in the current experiment on- and offsets of the duration were, on purpose, not clearly defined as distinct events, so one possible explanation of the higher variance could be that participants encountered difficulties in determining or remembering the exact on- and offset of an action. However, in the additional experiment (see Supplemental Material available at: https://osf.io/y9zex) we found that participants in general have a very precise and consistent idea of when an action started and ended, and how long it lasted. To shed more light on this matter, a future experiment could experimentally manipulate whether distinctiveness of on- and offsets has an effect on how well the interval marked by those events can be reproduced.

**Context Effects.** A consistent finding in studies on interval perception that use multiple, slightly different interval durations is the context effect. The duration of the previous trial (i.e. local context) and even the complete history of previously encountered interval durations within an experiment or experimental block (i.e. global context) affect the perception of the current interval (for a review, see van Rijn, 2016). Data from the current study exhibited similar effects of temporal context. Figure 3A shows the typical pattern of global context effects: shorter durations are systematically overestimated while longer durations are underestimated.

The analysis of local context effects revealed that not the *objective* duration of the previously presented stimulus, but the *subjective* previous duration influences our current interval perception. Since the previous subjective duration was in fact the previous interval reproduction, it may be a better measure of how participants perceived and stored a given interval than the objectively measured duration of the previous interval. Furthermore, any given subjective N-1 trial comprises the subjective N-2 trial (the N-1 of the N-1 trial), which itself comprises the subjective N-3 trial, and so on. Thus, subjective N-1 reflects the complete history of encountered intervals – the temporal context (see also Dyjas, Bausenhart, & Ulrich, 2012). These results demonstrate that in naturalistic tasks, empirical estimates of previously perceived durations, instead of objective duration, is a more powerful predictor of subsequent behaviour.

**Scalar Property.** Data from the current study is in conformity with the scalar property of time perception. We found that variation in interval reproduction linearly increased with interval duration, and that the coefficient of variation did not differ across interval durations (see Figure 3). Thus, participants’ timing sensitivity remained constant as the action durations varied from trial to trial. In principle, violations of, or large variations in, the coefficient of variation can hint at participants using counting strategies while performing interval timing tasks (Allman et al., 2014; Wearden & Lejeune, 2008). This was not the case in the current study. These results indicate that even though the onsets and offsets might be more difficult to perceive, timing sensitivity is stable and will play a similar role in ecologically valid settings as it does in laboratory studies.

**Perspective.** The present data showed no sign of an effect of first- versus third-person perspective. A possible explanation is that perceiving an action (or more generally: a scene) from first-person perspective is not sufficient to induce a feeling of authorship (Ebert & Wegner, 2010) or embodiment. That is, in our experiment, participants only passively viewed actions being performed – they did not perform actions themselves. Although the Bayes Factor provided decisive evidence against an effect of perspective in our setup, we prefer to refrain from making strong claims about general effects of perspective (see also the introduction) until methods that elicit stronger effects of perspective (i.e. paradigms utilizing virtual reality) have been tested.

**Conclusion.** Timing is extremely important in many tasks and settings, and this study demonstrates that deviations to veridical timing as observed in the laboratory also affect timing in the real world. We established that findings from artificial laboratory studies can be generalized to more real-world like settings and highlighted that, as in many domains, any percept is influenced by and embedded in context. Taken together, this study paves the way for studying interval timing as a component of complex, everyday cognitive performance.

## Data Accessibility Statement

All scripts to run the experiments and analyses as well as all data can be accessed at https://doi.org/10.17605/OSF.IO/Y9ZEX.
